# Seroprevalence of hepatitis B surface antigen and anti HCV antibody and its associated risk factors among pregnant women attending maternity ward of Felege Hiwot Referral Hospital, northwest Ethiopia: a cross-sectional study

**DOI:** 10.1186/s12985-015-0437-7

**Published:** 2015-12-02

**Authors:** Sefinew Molla, Abaineh Munshea, Endalkachew Nibret

**Affiliations:** Biology Department, Faculty of Natural and Computational Science, Debre Tabor University, Debra Tabor, Ethiopia; Biology Department, College of Science, Bahir Dar University, P.O. Box-79, Bahir Dar, Ethiopia

**Keywords:** HBV, HCV, Pregnancy, Seroprevalence, Risk factor

## Abstract

**Background:**

Viral hepatitis is a life-threatening liver disease that has become important public health issue in developing countries including Ethiopia. This study was undertaken to determine the seroprevalence of HBsAgs and anti-HCV antibodies and what socio-demographic factors are associated with sero-positivity of Hepatitis B Virus (HBV) and Hepatitis C Virus (HCV) infections among pregnant women attending maternity ward of Felege Hiwot Referral Hospital, northwest, Ethiopia.

**Methods:**

Hospital based cross-sectional study was conducted from November 2013 to January 2014. Blood samples were randomly collected from 384 pregnant women. Data on socio-demographic characteristics, obstetric and potential risk factors were collected using semi-structured questionnaire. Chromatographic kits were used to detect the presence of HBsAg and antibodies against HCV in serum samples of the studied subjects. Chi-square test was used for assessing the association between socio-demographic variables and HBV and HCV status. Logistic regression analysis was done to determine the strength of association between risk factors and HBV or HCV infection. *P-values* less than 0.05 were considered as significant.

**Results:**

Seroprevalnce of hepatitis B and C virus infections were found to be 4.4 and 0.26 %, respectively. None of the pregnant women were co-infected by these two viruses. Amongst the potential risk factors, previous history of dental procedure (AOR = 4.104, CI = 1.276–13.201, *P =* 0.018), house hold contact (AOR = 5.475, CI = 1.472–20.368, *P* = 0.011), multiple sexual exposure (AOR = 5.041, CI = 1.580–16.076, *P* = 0.006), and delivery at traditional birth attendants (AOR = 4.100, CI = 0.195-86.129, *P* = 0.024) were significantly associated with and important predictors of hepatitis B infection.

**Conclusions:**

This study found an intermediate endemicity (4.4 %) of HBV infection in pregnant women whereas seroprevalence of anti-HCV antibody was very small, but this needs to be confirmed by other similar studies with larger sample size. Thus, scaling up of the screening of pregnant women for HBV and HCV infections and provision of health education about the risk factors, the mode of transmissions and prevention is recommended.

## Introduction

Hepatitis is the inflammation of liver, most commonly caused by viral infections. Five hepatotropic viruses (A to E) are known to cause hepatitis. Of these, hepatitis B virus (HBV) and hepatitis C virus (HCV) are of greater importance and among the most frequent viral infections in humans [1, 2]. Globally, there are about 240 million people with chronic HBV infection and 130 to 150 million people with chronic HCV infection, reaching endemic proportions in sub Saharan Africa. HBV is estimated to result in 780,000 deaths and HCV in 350,000 to 500,000 deaths annually [3, 4].

HBV is highly contagious and relatively easy to be transmitted from one infected individual to another by blood to blood contact, during birth, unprotected sex, and by sharing needles while, HCV is transmitted mainly by parenteral routes such as intravenous drug use or blood product transfusion, transmission during sexual contact or during delivery is also possible, but is much less common [3, 4].

Chronic infection with HBV and HCV are often asymptomatic, but can lead to liver cirrhosis and hepatocellular carcinoma. Thus, most infected people are unaware of their HBV or HCV statuses until significant liver damage has occurred. Severe liver diseases are more frequent when patients are coinfected by the two viruses [5]. Combined HBV/HCV infection is sometimes observed because of the overlap in transmission routes of these viruses [6].

Viral hepatitis during pregnancy is associated with high risk of maternal, fetal and neonatal complications. There is a high rate of vertical transmission of HBV causing fetal and neonatal hepatitis, which may lead to impaired mental and physical health later in life [7]. Neonatal hepatitis can lead to chronic virus carriage, which in turn may lead to liver cirrhosis and hepatocellular carcinoma in young adults [7, 8]. Apart from this, acute hepatitis in pregnancy has been shown to induce premature labor and prematurity with its attendant effects [9, 10].

Incidence of hepatitis varies greatly around the world. In developed countries, the incidence is around 0.1 % whereas in developing countries it can range from 3 to 20 % or higher. There is no difference in the course of the disease in pregnant and non-pregnant women in developed countries. However, in developing countries, there is a higher incidence of maternal mortality with fulminant hepatitis [11]. This difference may be attributable to the variation in population studied, genetic factors, socioeconomic status, cultural practices and regional differences in risk factors to viral hepatitis [12].

HBV and HCV infections are the major global public health problems spreading rapidly in the developing countries including Ethiopia. Hence, screening antenatal women for hepatitis B surface antigen and anti HCV antibodies can give a reliable prevalence of the disease in a population and provide an avenue for preventing mother to child transmission of the virus. To the best of our knowledge, an epidemiological report of this type is sparse and quite inadequate in the country. Thus, the current study was aimed at investigating the seroprevalence and the possible risk factors of HBV and HCV among pregnant women attending maternal care Bahir Dar, Felege Hiwot Referral Hospital, northwest, Ethiopia.

## Materials and methods

### Study design and period

A hospital based cross sectional study was conducted from November 2013 to January 2014 so as to determine seroprevalence of HBsAgs and anti HCV antibodies and associated risk factors among pregnant women attending antenatal care center of Felege Hiwot Referral Hospital, Bahir Dar, Ethiopia.

### Source and study population

All pregnant women attending antenatal care center of Felege Hiwot Referral Hospital were considered as a source population while, those who visited the antenatal care center of the hospital during sample collection period were considered as a study population.

### Inclusion and exclusion criteria

Pregnant women who were willing to participate and provide consent were included in the study. Those who were already positive for hepatitis B and C and who did not consent to the study were excluded.

### Study variables

#### Dependent variables

In this study, two dependent variables, serostatus of hepatitis B and C viruses of respondents were studied.

#### Independent variables

Socio-demographic characteristics like, age, residence, educational, occupational status, income and marital status and clinical factors like parity status and gestational age were studied. History of blood transfusion, history of abortion, surgical procedure, dental surgery, tattooing, unsafe injection, history of house hold contact, multiple sexual exposure, use of condom and mode of delivery were also examined as possible risk factors for HBV and HCV.

### Sampling technique and sample size estimation

The study participants were selected by simple random sampling method based on the random accessibility of the patient during the study period until the required sample size was obtained. In the estimation of the sample size, statistical formula for sample size calculation was considered as a basis [13]. As prevalence of these infectious diseases is not known in the study area, the sample size of the proposed study was calculated using 50 % prevalence and a minimum sample size of 384 was obtained and included in the present study.

$$ n=\frac{Z^2}{d^2}P\left(1-P\right) $$

Where *n* = sample size

d = margin of error (5 %)

P = prevalence (50 %)

z = critical value at 5 % level (1.96)

### Data collection and processing

#### Socio-demographic characteristics and potential risk factors

Study participants who fulfilled eligibility criteria were interviewed by the nurses to gather relevant information using pre-designed questionnaire. The questionnaire included socio-demographic characteristics like age, residential area, and level of education, occupation, income, marital status, parity status and gestational age. Data on potential risk factors like history of dental and surgical procedure, tattooing, exposures to unsafe injection, history of delivery at traditional birth attendants, abortion, and history of house hold contact, blood transfusion and ear piercing in jewelers shop were also obtained.

#### Blood sample collection and processing

Five milliliters of venous blood were collected by medical laboratory technicians and the blood samples were left for 30 min to facilitate clotting. Then the clotted blood samples were centrifuged to separate the serum from blood cells. The serum was divided in two aliquots: one for HBsAg and the other for Anti-HCV antibody screening.

#### Serological detection of hepatitis B surface antigen and Anti-HCV test

Advanced Quality One Step HBsAg test strip was used for the detection of hepatitis B surface antigen (HBsAg) following the instructions of the manufacturer (Intec Products, Inc. (Xiamen), China). For the detection of antibodies produced against HCV dBest one Step HCV test strip was used following the instructions of the manufacturer (Ameritech Diagnostic Reagent Co, Tongxiang, Zhejiang, China). The sensitivity and specificity of the HBV test were 98.89 and 98.87 % respectively, whereas for the anti-HCV test they were 93.3 and 99.5 % respectively.

### Data analysis and interpretation

The prevalence of each viral infection (HBV and HCV) was determined from the proportion of the positive individuals in the total population under consideration and expressed as a percentage. The collected data were entered and analyzed using SPSS statistical software (version 20.0). They were organized and summarized in terms of frequencies and the results of the study were presented in tables. The chi-square (*χ*^2^) test was utilized for assessing statistical significance of association that could exist between socio-demographic variables and HBV and HCV status and *p-*values less than 0.05 were considered as significant. Logistic regression analysis was done to determine the strength of association between risk factors and HBV or HCV infection.

### Ethical considerations

Ethical clearance was obtained from ethical review board of Bahir Dar University. After explaining the objective of the study, first verbal informed consent and then written consent was obtained from each of the study participants to gather blood samples and data on sociodemographics. Subjects positive for HBsAg and anti-HVC antibody were reported to physicians for possible follow up and treatment. All information collected during the study was kept confidential.

## Results

### Socio demographic and clinical data of study participants

A total of 384 pregnant women were included in this study from November 2013 to January 2014. The mean age of the study subjects was 26.96 years (SD ± 4.58) with majority of the women falling in the age category of 25 to 29 years. This age category constituted 45.8 % of the total pregnant women and the lowest number of pregnant women was in15–19 years (1.3 %). Majority of the subjects were married (93.2 %) followed by single (5.5 %) and separated (1.3 %). Of participants, 43 (11.2 %) did not attend formal education while 103 (26.8 %) could read and write. Among the 384 pregnant women, 340 (88.5 %) were urban residents and 44 (11.5 %) were from rural settings (Table 1).

**Table 1:** Socio demographic characteristics of the study participants

Parameter		Frequency	Percent
		*n* = 384	(%)
Age group (years)	15–19	5	1.3
	20–24	108	28.1
	25–29	176	45.8
	30–34	62	16.1
	35–39	26	6.8
	40–44	7	1.8
Marital status	Married	358	93.2
	Single	21	5.5
	Separated	5	1.3
Residential area	Urban	340	88.5
	Rural	44	11.5
Educational Status	Illiterate	43	11.2
	Read and write	103	26.8
	Secondary school	116	30.2
	College and above	122	31.8
Occupation	Daily laborer	17	4.4
	House wife	162	42.2
	Privately employed	83	21.6
	Gov’t employee	111	28.9
	Other	11	3.4
Monthly income (Birr)	<500	74	19.3
	500–1000	88	21.6
	>1000	222	59.1

### HBV and HCV seroprevalence and socio- demographic and clinical characteristics

In this study, about 18 pregnant women (4.7 %) had serological evidence of infection with viral hepatitis, of these, the seroprevalence of HBsAg and anti HCV antibody was 4.4 and 0.26 %, respectively (Table 2) and while no co-infected pregnant women were detected.

**Table 2:** Seroprevalence of hepatitis B and C infections among the study subjects

Variables	Status	Total (%)
HBsAg	Positive	17 (4.4)
	Negative	367 (95.6)
Anti –HCV antibody	Positive	1 (0.26)
	Negative	383 (99.74)
Co-infection (HBsAg & HCV)	Positive	0 (0)
	Negative	384 (100)
Total		384 (100)

### Association of HBsAg sero-positivity and socio-demographic characteristics

HBsAg sero-positivity was detected in most of age categories except for 15–19 and 35–39 years old. The highest age-specific prevalence was identified in the age group 40–44. Out of seven women screened in this age category, 14.3 % tested positive for HBsAg. There was no statistically significant association between distribution of HBsAg and the age categories (*P* = 0.23). Majority of the study participants (93.2 %) were married, of which 4.2 % were positive for HBsAg. From 21 single pregnant women two (9.5 %) were positive for HBsAg and none of the separated women tested positive for HBsAg. There was no statistically significant association between HBsAg and the marital status of the study subjects (*P* = 0.456). The prevalence of HBV infection in relation to residence revealed that of the total 340 urban and 44 rural dwellers, 4.7 and 2.3 %, respectively, tested positive for HBsAg. However, no statistically significant association was detected between seroprevalence of HBsAg and residence (*P* = 0.477) (Table 3).

**Table 3:** Seroprevalence of HBsAg in relation to socio demographic characteristics

Parameter	Level	HBV status				Df	Chi-square	*p*-value
		Negative		Positive				
		Count	Percentage	Count	Percentage			
Age (years)	15–19	5	100.0	0	0.0			
	20–24	106	98.1	2	1.9			
	25–29	167	94.6	9	5.1			
	30–34	56	91.9	5	8.1	5	6.872	0.23
	35–39	27	100.0	0	0.0			
	40–44	6	85.7	1	14.3			
Marital status	Married	343	95.8	15	4.2	2	1.568	0.456
	Single	19	90.5	2	9.5			
	Separated	5	100.0	0	0.0			
Residential area	Urban	324	95.3	16	4.7	1	0.505	0.477
	Rural	43	97.3	1	2.3			
Educational status	Illiterate	42	97.7	1	2.3	3	0.96	0.811
	Read and write	97	94.2	6	5.8			
	Secondary school	111	95.7	5	4.3			
	College and above	117	95.9	5	4.1			
Occupation	Daily laborer	16	94.1	1	5.9			
	House wife	158	97.5	4	2.5	4	2.87	0.58
	Privately employee	78	94.0	5	6.0			
	Gov’t employee	105	94.6	6	5.4			
	Others	10	90.9	1	9.1			
Monthly income	<500	69	93.2	5	6.8			
	500–100	83	94.3	5	5.7	2	2.128	0.345
	>1000	215	96.8	7	3.2			
Parity status	Primigravidae	163	97.6	4	2.4	2	3.385	0.184
	Gravidae two	112	94.9	6	5.1			
	Multigravidae	92	92.9	7	7.1			
Gestational Age	First trimester	39	100.0	0	0.0			
	2nd trimester	126	92.6	10	7.4	2	5.13	0.077
	3rd trimester	202	96.7	7	3.3			

Occupation wise, the highest (9.1 %) frequency of HBV infection was observed among women who are involved in different activities followed by those who are privately employed (6.0 %). In this study, no statistically significant association was observed between HBsAg positivity and occupation of the study subjects (*P* = 0.58). Relatively, similar rates of serological evidence of HBsAg were detected among most educational categories except for those who are illiterate (2.3 %). However, there is no statistically significant association between HBsAg distribution and educational status (*P =* 0.811). The highest seroprevalence (6.8 %) of HBsAg was among women who earn the lowest monthly income (<500 Ethiopian birr/month). But, no statistically significant association was observed between seroprevalence of HBsAg and monthly income (*P* = 0.345). The prevalence of HBV infection among women who were pregnant for the first time was 2.4 %, while it was 5.1 % among the gravidae II and 7.1 % among multi- gravidae. However, no statistically significant relation was detected between HBsAg distribution and parity status of study subjects (*P =* 0.184). 7.4 and 3.3 % of pregnant women in their second and third trimester respectively tested positive for HBsAg, whereas, none of those in the first trimester infected with HBV. However, occurrence of HBsAg and gestational age was not statistically significant (*P* = 0.077) (Table 3).

### Potential risk factors of HBV and HCV among the study subjects

Associations between HBV infection and risk factors were analyzed using logistic regression. Multivariate logistic regression analysis was used for controlling confounders and for evaluating the strength of association of risk variables with HBV infection among studied group. The variables (dental extraction, history of delivery by the help of traditional birth attendants, history of household contact and history of multiple sexual exposures) were found to be associated with HBV infection in the univariate analysis were entered into the multivariate logistic regression model. In the final model, the same four variables were found to be significant predictors of HBV infection (*p* < 0.05) (Table 4).

**Table 4:** Association of potential risk factors and hepatitis B virus infection among study subjects

Variables	HBV status		COR(CI)	AOR(CI)	*P*-value
	Positive *N* (%)	Negative *N* (%)			
History of abortion					
Yes	4(4.3)	91(95.7)	0.933(0.297–2.934)	0.528(0.135–2.06)	0.358
No	13(4.5)	276(95.5)	1	1	
Surgical operation					
Yes	1(2.2)	45(97.8)	0.447(0.058–3.454)	0.367(0.035–3.822)	0.402
No	16(4.7)	322(95.3)	1	1	
Blood transfusion					
Yes	1(11.1)	8(89.9)	2.805(0.331–23.798)	2.136(0.177–25.735)	0.550
No	16(4.3)	359(95.7)	1		
Ear piercing					
Yes	10(4.6)	207(95.4)	1.097(0.409–2.946)	0.532(0.171–1.657)	0.276
No	7(4.2)	160(95.8)	1	1	
Exposure to unsafe injection					
Yes	3(8.1)	34(91.9)	2.099(0.574–7.67)	3.288(0.727–14.875)	0.122
No	14(4.0)	333(96.0)	1	1	
Dental extraction					
Yes	11(9.7)	102(90..3)	4.763(1.376–10.014)	4.104(1.276–13.201)	0.018*
No	6(2.2)	265(97.8)	1	1	
History of house hold contact					
Yes	5(20)	20(80.0)	7.229(2.320–22.524)	5.475(1.472–20.368)	0.011*
No	12(3.3)	347(96.7)	1	1	
History of multiple sexual exposure					
Yes	12(9.5)	114(90.5)	5.326(1.833–15.473)	5.041(1.580–16.076)	0.006*
No	5(1.9)	253(98.1)	1	1	
Delivery by traditional birth attendants					
Yes	12(7.2)	155(92.8)	3.794(0.481–29.917)	4.100(0.195–86.129)	0.024*
No	1(2.0)	49(98.0)	1	1	

Key: *COR* crude odd ratio, *AOR* adjusted odd ratio

*p-value <0.05, 1 = reference value

In relation to dental extraction, 29.2 % of the study subjects responded that they had previous history dental extraction, of which, 9.7 % were found to be positive for HBsAg. Statistically significant association was observed between previous history dental extraction and HBV infection (*P* = 0.018). Those who had dental extraction were 4.034 times more likely to have infection with HBV than their counterparts (AOR = 4.104, 95 % CI = 1.276–13.201).

Pregnant women who had previous history of house hold contact were 6.3 %, of which 20 % were positive for HBsAg. Statistically significant association was detected between having previous contact with members of the house hold with HBV infection (*P* = 0.011). Pregnant women who had previous history of house hold contact were 5.451 times more likely to have infection with HBV than those without previous history of house hold contact (AOR = 5.475, 95 % CI = 1.472–20.368).

The proportion of study participants who had multiple sexual partners was 32.8 %, of which 9.5 % were positive for HBsAg. Statistically significant association was detected between HBV infection and having multiple sexual partner (*p* = 0.006). Women having history of multiple sexual partner were 5.168 times at elevated risk of contracting HBV infection (AOR = 5.041, 95 % CI = 1.580–16.076) (Table 4).

Among 384 pregnant women, 167 had a history of delivering at home by traditional birth attendants, of these, 7.2 % were positive for HBsAg. Statistically significant association was obtained between these variables (*p* = 0.024). Those pregnant women with history of home delivery by traditional birth attendants were 4.100 times more likely to have HBV infection (AOR = 4.100, 95 % CI = 0.195–86.129).

## Discussion

HBV and HCV infections are significant health problems around the globe. Both infections are associated with a broad range of clinical presentations ranging from acute hepatitis to chronic infection that may be clinically asymptomatic or may progress to chronic hepatitis and liver cirrhosis [3, 4]. HBV infection affecting pregnant women may result in severe disease to the mother and chronic infection to the newborn [8]. The long term morbidity and mortality of HCV is far greater than HBV [14]. It has been reported that infants born to women with hepatitis C infection to be at risk of poor birth outcomes, including preterm birth, low birth weight, and congenital anomaly [15].

In the present study, sera collected from pregnant women were screened for HBsAg and anti-HCV. HBsAg detection serves as a marker for active HBV infection and infectivity whereas anti-HCV is a marker that shows someone has been exposed to HCV previously. The seroprevalence of hepatitis B and hepatitis C viral infections among the present study participants was 4.4 and 0.26 %, respectively. HBsAg is the main marker indicating prevalence as well as endemicity of HBV infection in the general population of particular geographical area [16]. On the basis of the carrier rate of this maker, WHO categorized countries of the world in to 3 regions of high (>8 %), medium (2–7 %) and low endemicity (<2 %) [17]. The 4.4 % prevalence of HBsAg observed in the present study area appears to be an intermediate endemicity according to WHO criteria of global epidemiology of HBV infection [17, 18].

The seroprevalence of hepatitis B surface antigen in this study is in concordance with the study carried out in Dar es Salaam, Tanzania 3.9 % [19], in Egypt 4.0 % [20] and in Port Harcourt, South Nigeria 4.3 % [21]. Furthermore, the present result is in line with the findings of similar studies from two Asian countries, Saudi Arabia (4.1 %) [22] and Pakistan (4.6 %) [23]. However, the prevalence of HBsAg in this study is lower than prevalence rates of 7.9, 9.3 and 10.2 % reported among similar antenatal clinic attendees in Kano, Nigeria [24], in Kenya [25] and in Far North Region of Cameroon [26], respectively. Likewise, it was also lower compared to recent similar studies reported from different parts of the world [27–29]. In contrast, HBsAg seroprevalence in this study is relatively higher than the findings of studies from 0.61 % India [30], 1.5 % Libya [31], 1.53 % Afghanistan [32] and 1.65 % Mexico [33]. Differences in demographics, cultural practices and behavior of the study population for the risk of HBV infection might explain these discrepancies.

The seroprevalence of anti-HCV antibody in the present study was found to be 0.26 % (1/384). This result was nearly similar with a study conducted in Nigeria 0.6 % [34]. In contrast, the finding in this study was much lower than anti HCV antibody seroprevalence rates of 3.6 and 8.5 % demonstrated in Nigeria [27] and Yemen [28], respectively. The lower prevalence of anti HCV antibody in the current study might be due to the difference in habit of using intravenous drug, exposure to blood transfusion, difference in efficiency of diagnostic kits used.

Comparable studies from different countries have documented coinfection of HBV and HCV [12, 27], however, no coinfection of these viral infections were detected in our study, which is in line with the findings of Murad et al. [28] and Oladeinde et al. [35].

In the present study, socio-demographic variables like age, marital and educational status, residence and occupations of participants as well as reproductive variables like gravidity and parity were not significantly associated with the risk of HBV infection (*P* > 0.05). This result is consistent with the reports by Rabiu et al. [36] and Oladeinde et al. [37]. However, it is difficult to rule out the associations of these variables totally with HBV infection.

In multivariate analysis, history of delivery by a traditional birth attendant (TBA) was found to be significantly associated with HBV infection (*P* = 0.024). Pregnant women who delivered by TBAs were 4.100 times more likely to have HBV infection. This is in agreement with a report from Nigeria [35]. Repeated use of old and unsterilized instruments during delivery by uneducated and unskilled birth attendants may account for this observation [38]. Therefore, it is plausible to suggest that engagement in these activities could have exposed pregnant women to HBV infection.

It has been reported that HBV can be transmitted between family members within a household through sharing personal items such as, tooth brushes and shaving razors with an infected person and exposure to blood from needle sticks or other sharp instruments contaminated with HBsAg of chronically infected persons [39, 40]. In our study, statistically significant association was observed between subjects who had history of contact with HBV infected family member and HBV infection (*p* = 0.011). Pregnant women who had contact with HBV infected person in their household were 5.451 times more likely to be HBsAg seropositive. The habit of sharing various personal and household articles within the home, as demonstrated in this study, provided an important mechanism for the transfer of HBV from asymptomatic carriers to other family members [41].

Furthermore, dental treatment was found to be a potential risk factor for HBV infection in our study and pregnant women who had history of dental treatment were at 4.034 times increased risk of HBV infection. This finding is consistent with previous serologic data of pregnant women from Jimma, Ethiopia [42]. Likewise, Pande et al. [43] also evidenced that history of dental treatment was a risk factor for HBV infection. This might be due to non adherence to guidelines on infection control and use of non disposable or reusable equipments and the lack of sufficient sterilization technology.

Sexual transmission has long been recognized as a major source of HBV transmission in the world [44]. It is, therefore, not surprising to identify multiple sexual exposures as significant risk factor for HBsAg seropositivity. In the current study, statistically significant association was observed between history of multiple sexual exposure and HBV infection (*P* < 0.05). Individuals who had multiple sexual partners were at five fold of elevated risk of acquiring HBV infection. This is in agreement with the findings of Rabiu et al. [36] and Obi et al. [45], who also demonstrated a history of multiple sexual partners to be significant risk factors for HBV infection during pregnancy. Changes in sexual practice and behavior modification may therefore be an important step towards reduction of hepatitis B infection.

## Conclusions

An intermediate and low prevalence rates of HBsAg and anti HCV antibodies respectively, were detected in this study. However, no co-infection of these hepatotropic viruses was obtained. No statistically significant association was observed in acquisition of HBV infection and socio-demographics and variables like parity and gestational age. Previous history of dental treatment, household contact, delivery at traditional birth attendant and multiple sexual exposures were found to be important indicators of HBV infection in this study. There should be routine screening of all pregnant women for hepatitis B and C virus infections for possible early immunization of infants of seropositive mothers. Molecular characterization and detection of the other markers should be conducted to determine the exact prevalence of HBV and HCV in future study.

## References

[1] El-Serag HB (2012). Epidemiology of viral hepatitis and hepatocellular carcinoma. Gastroenterology.

[2] Eke AC, Eke UA, Okafor CJ (2011). Prevalence correlates and pattern of hepatitis B surface antigen in a low resource setting. Virol J.

[3] World Health Organization. Hepatitis C: Factsheet No. 164. 2015. http://www.who.int/topics/hepatitis/factsheets/en/. Accessed 10 Aug 2015.

[4] World Health Organization. Hepatitis B: Factsheet No. 204. 2015. http://www.who.int/mediacentre/factsheets/fs164/en/. Accessed 10 Apr 2015.

[5] Fattovich G, Stroffolini T, Zagni I, Donato F (2004). Hepatocellular carcinoma in cirrhosis: incidence and risk factors. Gastroenterology.

[6] Liu Z, Hou J (2006). Hepatitis B virus (HBV) and hepatitis C virus (HCV) dual infection. Int J Med Sci.

[7] Sookoian S (2006). Liver disease during pregnancy: acute viral hepatitis. Ann Hepatol.

[8] Wright TL (2006). Introduction to chronic Hepatitis B infection. Am J Gastroenterol.

[9] Bohidar NP (2004). Hepatitis B, virus infection in pregnancy. Hep B Annual.

[10] Gambarin-Gelwan M (2007). Hepatitis B, in pregnancy. Clin Liver Dis.

[11] Khuroo MS, Kamili S (2003). Aetiology and prognostic factors in acute liver failure in India. J Viral Hepat.

[12] Esan AJ, Omisakin CT, Ojo-Bola T, Owoseni MF, Fasakin KA, Ogunleye AA (2014). Sero-Prevalence of Hepatitis B and Hepatitis C Virus Co-Infection among Pregnant Women in Nigeria. Am J Biomed Res.

[13] Naing L, Winn T, Rusli BN (2006). Practical issues in calculating the sample size for prevalence studies. Arch Orofac Sci.

[14] Eriksen NL (1999). Perinatal consequences of hepatitis C. Clin Obstet Gynecol.

[15] Connell LE, Salihu HM, Salemi JL, August EM, Weldeselasse H, Mbah AK (2011). Maternal hepatitis B and hepatitis C carrier status and perinatal outcomes. Liver Int.

[16] Shepard CW, Simard EP, Finelli L, Fiore AE, Bell BP (2006). Hepatitis B virus infection: epidemiology and vaccination. Epidemiol Rev.

[17] World Health Organization. Hepatitis B vaccines. Weekly Epidemiological Record. 84(40):405-20. http://www.who.int/wer/2009/wer8440.pdf. Accessed 12 Feb 2015.

[18] Colin WS, Edgar PS, Lyn F, Anthony E, Fiore BP (2006). Epidemiology of Hepatitis B Virus Infection. Epidemiol.

[19] Rashid S, Kilewo C, Aboud S (2014). Seroprevalence of hepatitis B virus infection among antenatal clinic attendees at a tertiary hospital in Dar es Salaam, Tanzania. Tanzan J Health Res.

[20] Zahran KM, Badary MS, Agban MN, Abdel-Aziz NH (2010). Pattern of hepatitis virus infection among pregnant women and their newborns at the Women’s health center of Assiut University, Upper Egypt. Int J Gynaecol Obstet.

[21] Obi RK, Umeh SC, Okurede OH, Iroagba II (2006). Prevalence of hepatitis B virus infection among pregnant women in antenatal clinic in Port Harcourt, Nigeria. Afr J Clin Exper Microbiol.

[22] Bani I, Salih M, Mahfouz ME, Gaffar A, Elhassan I, Yassin AO (2012). Prevalence and risk factors of hepatitis B virus among pregnant women in Jazan Region- Kingdom of Saudi Arabia. J Biol Agric Healthcare.

[23] Taseer IU, Ishaq F, Hussain L, Safdar S, Mirbahar AM, Faiz SA (2010). Frequency of anti-HCV, HBsAg and related risk factors in pregnant women at Nishtar Hospital, Multan. JAMC.

[24] Yakasai IA, Ayyuba R, Abubakar IS, Ibrahim SA (2012). Sero-prevalence of hepatitis B virus infection and its risk factors among pregnant women attending antenatal clinic at Aminu Kano Teaching Hospital, Kano, Nigeria. J Basic Clin Reprod Sci.

[25] Okoth F, Mbuthia J, Gatheru Z, Murila F, Kanyingi F, Mugo F (2006). Seroprevalence of hepatitis B markers in pregnant women in Kenya. East Afr Med J.

[26] Noubiap JJN, Nansseu JRN, Ndoula ST, Bigna JJR, Jingi AM, Fokom-Domgue J (2015). Prevalence, infectivity and correlates of hepatitis B virus infection among pregnant women in a rural district of the Far North Region of Cameroon. BMC Public Health..

[27] Ugbebor O, Aigbirior M, Osazuwa F, Enabudoso E, Zabayo O (2011). The prevalence of hepatitis B and C viral infections among pregnant women. N Am J Med Sci.

[28] Murad EA, Babiker SM, Gasim GI, Rayis DA, Adam I (2013). Epidemiology of hepatitis B and hepatitis C virus infections in pregnant women in Sana’a, Yemen. BMC Preg Childbirth.

[29] Bakthavatchalu S (2012). Hepatitis B, surface antigen carrier state among asymptomatic pregnant women and its correlation with vertical transmission. Jain University, Bangalore, India. Int J Res in Pharm and Sce.

[30] Shazia-Parveen S, Shyamala R, Janardhan-Rao R, Rama-Rao MV (2012). Seroprevalence of hepatitis B surface antigen among pregnant women attending antenatal clinic in a teaching hospital, Bhaskar Medical College, Ranga Reddy Dist, Andhra Pradesh, India. J Microbiol Biotech Res.

[31] El-Magrahe H, Furarah AR, El-Figih K, El-Urshfany S, Ghenghesh KS (2010). Maternal and neonatal seroprevalence of hepatitis B surface antigen (HBsAg) in Tripoli, Libya. J Infect Dev Ctries.

[32] Todd CS, Ahmadzai M, Atiqzai F, Miller S, Smith JM, Ghazanfar SA (2008). Sero-prevalence and correlates of HIV, syphilis, and hepatitis B and C virus among intrapartum patients in Kabul Afghanistan. BMC Infect Dis.

[33] Vazquez-Martinez JL, Coreno-Juarez MO, Montano-Estrada LF, Attlan M, Gomez-Dantes H (2003). Sero-prevalence of hepatitis B in pregnant women in Mexico. Salud Publica Mex.

[34] Buseri FI, Seiyabo E, Jeremiah ZA (2010). Surveying infections among pregnant women in the Niger Delta, Nigeria. J Global Infect Dis.

[35] Oladeinde BH, Omoregie R, Olley M, Anunibe JA, Oladeinde OB (2012). Hepatitis B and C viral infections among pregnant women in a rural community of Nigeria. Int J Basic and Applied Virol.

[36] Rabiu KA, Akinola OI, Adewunmi AA, Omololu OM, Ojo TA (2010). Risk factors for hepatitis B virus infection among pregnant women in Lagos, Nigeria. Acta Obstetricia Gynecologica.

[37] Oladeinde BH, Omoregie R, Oladeinde OB (2013). Prevalence of HIV, HBV, and HCV infections among pregnant women receiving antenatal care in a traditional birth home in Benin City, Nigeria. Saudi J Health Sci.

[38] Sadoh AE, Ogungbe RO (2008). Multiple fractures and latrogenic burns in a Newborn due to unskilled Delivery: A case Report. Afr J Reprod Health.

[39] EL-Shabrawi M, Mohamed MF, Hamdi MSED, Ehab M, Khamiss SS, El-Karaksy H (2013). Prevalence of hepatitis B virus infection among Egyptian pregnant women-A single center study. Int J Tropical Dis Health.

[40] Oladele OO, Saheed S, Familua F, Adekunle OO, Olusola O, Samuel BO (2014). Sero-prevalence of Hepatitis B Surface Antigen and Antibody among Pregnant Women Attending a Tertiary Health Institution in Southwestern Nigeria. OSR-JDMS.

[41] Datta S (2008). An overview of molecular epidemiology of hepatitis B virus (HBV) in India. Virol J.

[42] Mohammed A, Solomon G-S (2005). Seroprevalence of HBsAg and its risk factors among pregnant women in Jimma, southwest Ethiopia. Ethiop J Health Dev.

[43] Pande C, Sarin SH, Patra S, Bhutia K, Mishra SK, Pahuja S (2011). Prevalence, risk factors and virological profile of chronic hepatitis B virus infection in pregnant women in India. J Med Virol.

[44] Hou J, Liu Z, Gu F (2005). Epidemiology and prevention of hepatitis B virus infection. Int J Med Sci.

[45] Obi SN, Onah HE, Ezugwu FO (2006). Risk factors for hepatitis B infection during pregnancy in a Nigerian obstetric population. J Obstet Gynaecol.

